# Supplementary material for: Cardiac Rehabilitation for Fontan Circulation Patients: A Systematic Review, and Meta-Analysis
Source: Medicina (Kaunas). 2024 Nov 5;60(11):1817. doi: 10.3390/medicina60111817 (PMC11596103; doi:10.3390/medicina60111817)
Supplement: Supplementary file 1 [file medicina-60-01817-s001.zip › medicina-3187377-supplementary.pdf]

SUPPELEMENTARY DATA

Search string

(Fontan OR Fontan-Kreutzer OR (Fontan AND procedure) OR (Fontan AND technique) OR (Fontan–Kreutzer AND procedure) OR (Fontan-Kreutzer AND technique)) **AND** ("cardiac rehabilitation" OR rehabilitation OR exercise* OR (exercise* AND capacity) OR (exercise* AND training) OR (exercise* AND intervention*) OR (exercise* AND therap*) OR (exercise* AND rehabilitation))

Table 1. Study characteristics. All included studies were characterised based on author, year of publication, study design, number of patients included in the study, type of exercise training, training sessions/week, time/session, training period, homebased vs supervised, and follow-up time.

**Author**	**Year of publication**	**Study design**	**Training group (n)**	**Control group (n)**	**Type of exercise training**	**Sessions/week**	**Time/session**	**Training period**	**Homebased vs supervised**	**Follow-up time**
Neidenbach	2023	Non blinded, RCT	20<br>2 dropouts	20<br>1 dropout	IMT	Daily	3 sets 30 reps	6 months	Homebased	6 months
Minamisawa	2001	Cohort	11	0	AET	2-3x	20-30 min	2-3 months	3 weeks supervised<br><br>2-3 months homebased	3 months
Opocher	2005	Cohort	10	0	AET	2x	30-45 min	8 months	3 weeks supervised<br><br>Followed by homebased	8 months
Fritz	2020	RCT	20	22<br><br>At 6 months follow-up control group started IMT till 12 months	IMT	Daily	3 sets 10-30 reps	6 months	Homebased, telephone supervised	6, 12, 18 months
Turquetto	2021	RCT	AET: 10<br><br>IMT: 10	12	AET or IMT	AET: 3x<br><br>IMT: daily	AET: 60 min<br><br>IMT: 3 sets 30 reps	4 months	AET: supervised<br><br>IMT: first 2 months 1x/wk. Last 2 months 1x/2wks.	4 months

Dulfer<br>Duppen<br>Duppen	2014<br>2015<br>2015	RCT	26	17	AET	3x	60 min	12 weeks	Supervised	12 weeks
Sutherland	2018	Randomised trial	17	0	AET + resistance training lower limb muscles	2x	60 min	8 weeks	Homebased (n=11)<br><br>Supervised (n=6)	8 weeks
Jacobsen<br>Jacobsen	2016<br>2018	Cohort	13<br>11	0	AET + resistance exercises	3-4x	45 min	12 weeks	Homebased	12 weeks<br>6 months
Dirks	2020	Cohort	18	0	AET (Cycling)+ IMT	AET: 3-6x<br>IMT: 6-7x	AET: 90 min total<br><br>IMT: 30 breaths	10 months	Homebased	4 months<br>10 months
Avitabile	2022	Cohort	20	0	Lower extremity focused exercise	3x	60 min	24 weeks	Hybrid: 1 month Homebased	24 weeks
Perrone	2022	Cohort	12	0	AET	3x	40 min	4 weeks	Homebased	4 weeks
Pyykkönen	2022	Cohort	16	0	AET + bodyweight exercises focused on lower limbs	1-2x	6-8 exercises	6 months	Homebased	6 months
Wu	2018	Cohort	11	0	IMT	5x	30 min	12 weeks	Homebased	12 weeks
Hedlund<br>Hedlund	2018<br>2018	Cohort with controls	30	25 (healthy controls)	AET	2x	45 min	12 weeks	Supervised	12 weeks<br>1 year
Wittekind	2018	Cohort	10	0	AET + low resistance high repetition strength training	2x	60 min	12 weeks	Supervised	12 weeks
Ait ali	2018	Cohort with controls	10	6	CRT	1x supervised	Supervised: 120 min<br>Homebased: 240±90min/wk	3 months	Supervised + homebased	3 months
Cordina	2013	Cohort with controls	6	5	High intensity total body resistance training focused on calf muscle	3x	60 min	20 weeks	Supervised	20 weeks, 12 month detrain
Longmuir	2013	Randomized trial	30	0	AET + resistance training (play	1x	90-120 min	12 months	Homebased	6, 12 and 24 months

					based physical activities)					
Brassard	2006	Cohort with controls	5	9 (7 healthy, 2 Fontan)	AET + resistance training	3x	20-30 min	8 weeks	Supervised (n=2)<br>Homebased (n=3)	8 weeks
Scheffers	2023	Randomized semi-cross-over-controlled trial	28<br><br>Started training immediately: 14	14<br><br>Start 6 weeks of control period, thereafter 12 weeks of exercise period	Leg focused high weight resistance training + high protein diet	3x	45 min	12 weeks	Supervised	6 weeks<br>12 weeks
Bano	2023	Cohort	5	0	AET + resistance training	Start: 2x<br>Increasing to: 5x	30-45 min	3 months	Supervised	12 weeks
Laohachai	2017	Cohort	23<br>4 dropouts	0	IMT	Daily	30 min	6 weeks	Homebased	6 weeks

Abbreviations: N= number of patients. RCT= randomised controlled trial, AET= aerobic exercise training, IMT= inspiratory muscle training. CRT: controlled respiratory training. Wk= week. Reps= repetitions.

Table 2. Patient characteristics. Table 2 provides the characteristics of patients included in the studies: age, sex, ventricle type, type of Fontan and age at Fontan completion.

**Study**	**Age (years),** mean (range)/mean ± SD	**Sex,** % female	**Ventricle type,** % left	**Fontan type (number of patients)**	**Age (years) at Fontan completion,** mean (range)/mean ±SD
Neidenbach, 2023	12,3±2,2	25%	45%	Extracardiac conduit: 37<br>Lateral tunnel: 4	27,5±9,6 (months)
Minamisawa, 2001	19±4	55%	82%	Direct atrial to pulmonary anastomosis without valve	14±6
Opocher, 2005	8,7±0,6	10%	70%	Total cavopulmonary connection	1,7±0,8
Dulfer, 2014<br>Duppen, 2015<br>Duppen, 2015	15±3<br>15±3<br>14,8±3,7	60%	69%	Extracardiac conduit: 20<br>Intra-atrial lateral tunnel: 21<br>Other: 2	3 (2.5-5)<br>3 (2.5-4)<br>3 (2.5-4.4)

Fritz, 2020	28.6 (24.7-36.5)	50%	86%	Atrioventricular connection: 8<br>Atriopulmonary connection: 9<br>Total cavopulmonary connection: 25	6.3 (4.0-9.9)
Turquetto, 2021	20 (15-25)	69%	78%	Extracardiac conduit: 24<br>Lateral tunnel: 8	8 (7-11)
Sutherland, 2018	Homebased: 15±2.7<br>Hospital based: 16±2.5	41%	Not reported	Extracardiac conduit: 15<br>Lateral tunnel: 1<br>Atriopulmonary connection: 1	Not reported,<br>for all patients last cardiac operation longer than 5 years before enrolment.
Jacobsen, 2016<br>Jacobsen, 2018	10 (8-12)<br>10,5 (9-12)	55%	43%	Extracardiac conduit: 12<br>Lateral tunnel: 2<br>Patent fenestration: 7	Not reported,<br>time since Fontan: 7,4±2,3
Dirks, 2022	16,5 (10-43)	39%	Not reported	Not reported	Not reported
Avitabile, 2022	15.6 ±1,7	50%	45%	Extracardiac conduit: 14<br>Lateral tunnel: 6	Not reported,<br>time since Fontan: 11.7±3,4
Perrone, 2022	24±2,5 (22-27)	50%	0%	Not reported	Not reported
Pyykkönen, 2022	14,5±2,6 (8-18)	37.5%	21%	Not reported	2.9±0,5
Wu, 2018	28.8 (25,7-45,5)	45%	73%	Extracardiac conduit: 1<br>Lateral tunnel: 7<br>Atriopulmonary connection: 3	7,8 (3,9-16,5)
Hedlund, 2018<br>Hedlund, 2018	14,2±3,2	47%	Not reported	Extracardiac conduit (non-fenestrated)	2.4 (1,1-6,4)
Wittekind, 2018	12±2,8 (7-18)	60%	50%	Extracardiac conduit: 5<br>Lateral tunnel: 5	At least 5 years prior to enrolment
Ait ali, 2018	17,5±3,8 (10,4-22,8)	13%	43%	Extracardiac conduit: 10<br>Intracardiac conduit: 1<br>Intracardiac tunnel: 2<br>Kawasahima: 1	3,69 (0,8-7,4)
Cordina, 2013	31±4	18%	82%	*Intervention*<br>Extracardiac conduit: 1<br>Atriopulmonary connection: 2<br>Total cavopulmonary connection: 3<br><br>*Control*<br>Extracardiac conduit: 1	Not reported,<br>time since last Fontan repair intervention: 21±1<br>control: 18±2

				Atriopulmonary connection: 2<br>Total cavopulmonary connection: 2	
Longmuir, 2013	9, 1(7,7-10,5)	41%	54%	Extracardiac conduit: 53<br>Lateral tunnel: 7<br>Bjork procedure: 1	2,9 (2,5-3,8)
Brassard, 2006	16±5	43%	100%	Not reported	Not reported
Scheffers, 2023	12.9 (10.5-15.7)	37%	37%	Intra-artrial later tunnel technique for total cavo-pumonary connection	2.8 (2,3-3,8)
Bano, 2023	19.5 (17.6–21.3)	20%	40%	Extracardiac conduit (fenestrated): 5	Not reported,<br>time since Fontan operation: 17,4 (13,8-18,6)
Laohachai, 2017	16±2 (12-20)	48%	57%	Extracardiac conduit (non-fenestrated)	5±2 (3-9)

Table 3. Quality assessment RCTS – Cochrane risk of bias tool 2

	D1	*D2*	*D3*	*D4*	*D5*	*Overall risk of bias*
Dulfer Duppen Duppen	Low risk	Some concerns	Low risk	Low risk	Low risk	Low risk
Fritz	Low risk	Some concerns	Low risk	Low risk	Low risk	Low risk
Neidenbach	Low risk	Some concerns	Low risk	Low risk	Low risk	Low risk
Turquetto	Low risk	Some concerns	Low risk	Low risk	Low risk	Low risk
Scheffers	Low risk	Some concerns	Low risk	Low risk	Low risk	Low risk

Table 4 Quality assessment Cochrane risk of bias tool 2. D1: bias arising from the randomization process. D2: bias due to deviations from intended intervention. D3: bias due to missing outcome data. D4: bias in measurement of the outcome. D5: bias in selection of the reported result.

Table 4. Quality assessment cohort studies - STROBE checklist

	Minimasiwa	Jacobsen	Sutherland	Hedlund	Laohchai	Longmuir	Opocher	Wu	Cordina	Ait ali	brassard	wittekind	Dirks	Avitabile	perrone	Pykkönen	Bano
**Title and abstract**																	
Design in title	x	x	x	x	x	x	x		x	x		x		x	x	x	
Abstract																	
**Introduction**																	
Background/rationales																	
Objective																	
**Methods**																	
Study design																	
Setting																	
Participants																	
Variables																	
Data sources/measurement																	
Bias	x	x		x	x	x	x	x	x	x	x	x	x	x	x		x
Study size	x	x	x	x			x		x	x	x		x	x	x	x	x
Quantitative variables																	
Statistical methods																	
**Results**																	
Participants		x					x	x		x							
Descriptive data															x		
Outcome data																	
Main results																	
Other analyses																	

**Discussion**																	
Key results																	
Limitations																	
Interpretation																	
Generalisability																	
**Other information**																	
funding	x						x			x					x		
Criteria met (%)	84%	84%	93%	89%	93%	93%	80%	91%	89%	80%	91%	93%	91%	89%	80%	93%	91%

Table 5 Quality assessment STROBE checklist. If the items from the STROBE checklist were not or not precisely prescribed in the article the item was marked with a X. Eventually the percentage of the total items that were well prescribed in the article was calculated, demonstrated as 'criteria met (%)'.

Table 5. Study observations. Table 5 provides all observations of the included studies regarding the effect of exercise training programs.

**Studies**	**Peak VO2**	**VE/VCO2 slope**	**Peak workload**	**Activity levels**	**Distance walked (6mwt)**	**Cardiac output (MRI or echo)**	**Cardiac biomarkers**	**Lung function**	**Improvement Lower limb muscle**	**Quality of life**	**Adverse events**
Neidenbach, 2023 (16)	=	=	=	-	-	-	-	^	-	-	No
Minamisawa, 2001 (17)	^	-	^	-	-	-	-	-	-	-	No
Opocher, 2005 (18)	^	-	-	-	-	-	-	-	-	-	No
Fritz, 2020 (19)	=	=	-	-	-	-	-	^ *	-	-	Yes
Turquetto, 2021 (20)	^	=	-	-	^	-	-	^	-	^	No
Dulfer, 2014 (21)<br>Duppen, 2015 (22)<br>Duppen, 2015(23)	=	=	-	-<br>=<br>-	-	=	=	-	-	^	-
Sutherland, 2018 (24)	=	-	=	-	^	-	-	-	-	^	No
Jacobsen, 2016 (25)	^	-	-	=	-	-	-	-	-	^	No
Jacobsen, 2018 (26)	=	-	-	-	-	-	-	-	-	^	-
Dirks, 2022 (27)	^	-	-	-	-	-	-	^	-	=	No

Avitabile, 2022 (28)	=	-	^	=	-	=	-	-	=	=	No
Perrone, 2022 (29)	^	=	-	-	-	-	^	-	-	-	no
Pyykkönen, 2022 (30)	=	^	^	^	-	-	-	-	=	-	No
Wu, 2018 (31)	=	=	^	-	-	=	-	^	-	=	No
Hedlund, 2018 (32)<br>Hedlund, 2018 (33)	=	=	=	^	^	-	-	^	-	^	-
Wittekind, 2018 (34)	^	^	^	-	-	=	-	-	-	-	No
Ait ali, 2018 (35)	^	=	=	-	-	-	-	^	-	-	-
Cordina, 2013 (36)	^	-	^	-	-	^	-	-	-	-	Yes
Longmuir, 2013 (37)	=	-	-	^	-	-	-	-	-	-	-
Brassard, 2006 (38)	=	=	=	-	-	-	-	=	=	-	-
Scheffers, 2023 (39)	^	-	^	=	^	^	=	-	^	^	Yes
Bano, 2023 (40)	^	=	-	-	-	=	-	-	-	^	No
Laohachai, 2017 (41)	=	^	=	-	-	^	-	^	-	-	No

*Abbreviations. 6mwt: six minute walking test. - : not reported. =: no significant improvement or decrease, ^: significant improvement. *: only the lung function oxygen saturation at rest improved.*
